# Supplementary material for: Enhancement of Immune Response of Bioconjugate Nanovaccine by Loading of CpG through Click Chemistry
Source: J Pers Med. 2023 Mar 11;13(3):507. doi: 10.3390/jpm13030507 (PMC10052328; doi:10.3390/jpm13030507)
Supplement: Supplementary file 1 [file jpm-13-00507-s001.zip › jpm-2263030-supplementary.pdf]

Supplementary Materials:

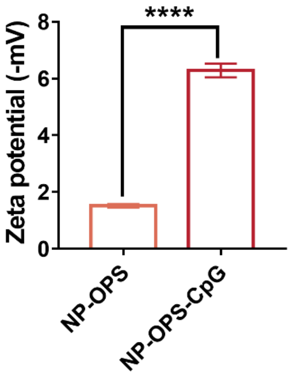

Figure S1. The zeta potential of NP-OPS and NP-OPS-CpG. Samples were analyzed by DLS. (n = 3) NP-OPS was compared with NP-OPS-CpG using t-test: *** $p < 0.001$.

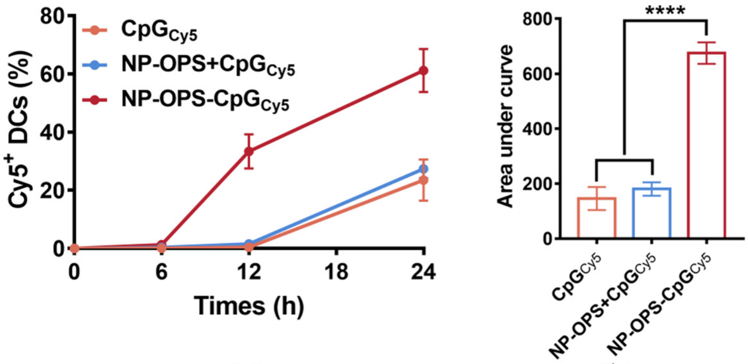

(**a**) (**b**)

Figure S2. Effeciency of DC2.4s phagocytosis NP-OPS-CpG. (**a**) The DC2.4s engulfed CpG curve. DC2.4s were analyzed for Cy5^{+} content at 6 h, 12 h, and 24 h after stimulation by NP-OPS-CPG$_{Cy5}$, NP-OPS+CpG$_{Cy5}$ and CpG$_{Cy5}$ (n = 3). (**b**) Accumulation of CpG (labeled by Cy5) in DC2.4s within 24 h (n = 3). Each group was compared with NP-OPS-CpG using one-way ANOVA: **** $p < 0.0001$.

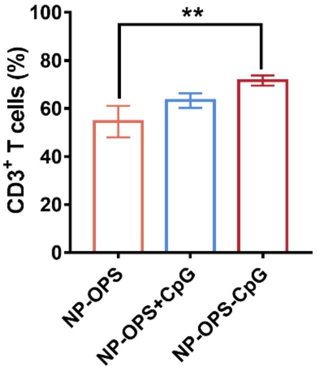

Figure S3. T cells immune responses induced by NP-OPS-CpG *in vivo*. Lymph nodes were obtained from mice five days after the third immunization and CD3^{+} cells were analyzed by flow cytometry (n = 3). Each group was compared with NP-OPS-CpG using one-way ANOVA: ** $p < 0.001$.
